# Absolute Quantification of Nav1.5 Expression by Targeted Mass Spectrometry

**DOI:** 10.3390/ijms23084177

**Published:** 2022-04-10

**Authors:** Sarah L. Adams, Ge Chang, Mohamed A. Fouda, Sharwan Kumar, Bingyun Sun

**Affiliations:** 1 Department of Molecular Biology and Biochemistry, Simon Fraser University, Burnaby, BC V5A 1S6, Canada; sarah_adams_2@sfu.ca (S.L.A.); sharwan_kumar_2@sfu.ca (S.K.); 2 Department of Chemistry, Simon Fraser University, Burnaby, BC V5A 1S6, Canada; ge_chang@sfu.ca; 3 Department of Biomedical Physiology and Kinesiology, Simon Fraser University, Burnaby, BC V5A 1S6, Canada; mohamed_fouda@sfu.ca

**Keywords:** targeted mass spectrometry, parallel reaction monitoring (PRM), sodium voltage-gated channel alpha subunit 5 gene (SCN5A) and its corresponding protein (Nav1.5), membrane protein expression, absolute protein quantification, protein copy numbers, cardiac diseases, cardiomyocytes, ion channels

## Abstract

Nav1.5 is the pore forming α-subunit of the cardiac voltage-gated sodium channel that initiates cardiac action potential and regulates the human heartbeat. A normal level of Nav1.5 is crucial to cardiac function and health. Over- or under-expression of Nav1.5 can cause various cardiac diseases ranging from short PR intervals to Brugada syndromes. An assay that can directly quantify the protein amount in biological samples would be a priori to accurately diagnose and treat Nav1.5-associated cardiac diseases. Due to its large size (>200 KD), multipass transmembrane domains (24 transmembrane passes), and heavy modifications, Nav1.5 poses special quantitation challenges. To date, only the relative quantities of this protein have been measured in biological samples. Here, we describe the first targeted and mass spectrometry (MS)-based quantitative assay that can provide the copy numbers of Nav1.5 in cells with a well-defined lower limit of quantification (LLOQ) and precision. Applying the developed assay, we successfully quantified transiently expressed Nav1.5 in as few as 1.5 million Chinese hamster ovary (CHO) cells. The obtained quantity was 3 ± 2 fmol on the column and 3 ± 2 × 10^4^ copies/cell. To our knowledge, this is the first absolute quantity of Nav1.5 measured in a biological sample.

## 1. Introduction

**Figure 1:**
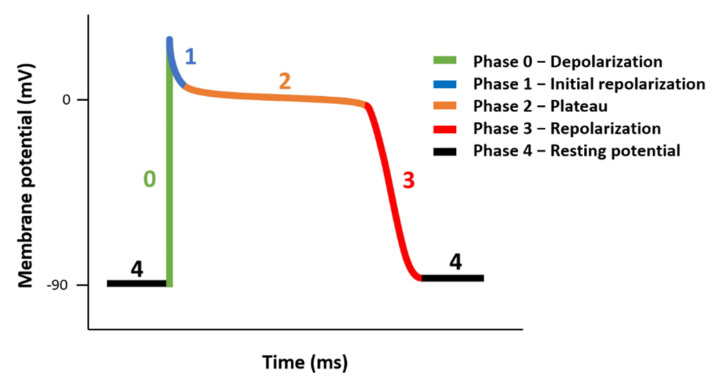
Illustrations of cardiac action potential.

Human cardiac voltage-gated sodium channel comprises an α-subunit and four β-subunits, β1–β4 [1,2]. The sodium voltage-gated channel alpha subunit 5 gene (SCN5A) gene encodes the pore forming α-subunit, Nav1.5 protein, which mediates the inward sodium current (I_Na_) [3,4,5] in cardiac muscle. The I_Na_ induces a rapid depolarization of membrane potential and triggers the cardiac action potential (AP), as shown in Figure 1, which in turn initiates the excitation–contraction coupling cascade in cardiomyocytes [3,4,5]. Defects in Nav1.5/SCN5A affect all aspects of cardiac function and can lead to torsade de pointes, syncope, ischemic cardiomyopathy, cardiac arrest, congenital cardiac arrhythmias, and lethality as a result of heart failure and sudden cardiac death [1,5,6]. For example, gain-of-function in Nav1.5 was observed in patients with long-QT syndrome (LQT3) [7,8,9], in which increased late I_Na_ causes a prolonged cardiac action potential [8,9]. Both gating and increased Nav1.5 surface expression can cause long-QT in mouse models [10,11]. However, human validation of these observations has been challenging due to ethical and experimental challenges. Furthermore, overexpression of Nav1.5 in normal mice resolved the shortened P wave and PR intervals that mimic enhanced atrioventricular nodal conduction in humans [12,13]. Conversely, decreased expression in Nav1.5 has been observed in patients with Brugada Syndrome (BrS) [14,15,16], in which the decreased I_Na_ triggered an epicardial reentrant excitation and an elevated ST segment in the electrocardiogram [1,14,16]. Other syndromes, such as sick sinus node syndrome, progressive cardiac conduction defects, and atrial fibrillation can also be observed in patients with altered Nav1.5 expression [5]. Therefore, the expression and function of Nav1.5 need to be tightly regulated in a finite range for normal cardiac physiology. Malfunctions in Nav1.5 have been frequently observed clinically through genetic testing of SCN5A mutations [5,6,8,17]. Little knowledge exists on acquired instead of inherited expression changes of Nav1.5 in cardiac health, even though most cardiac diseases, such as heart failure and myocardial ischemia, are acquired and have exhibited changes in Nav1.5 levels [18,19]. It is well known that the I_Na_ current and the function of the channel are directly affected by protein expression [6,20]. A reduction of Nav1.5 expression in mice with a heterozygous knockout (SCN5A^+/−^) reduced 50% of I_Na_ conduction and resolved ventricular tachycardia and conduction defects [21], which recapitulated clinical observations in humans. Using mathematical modeling of Nav1.5/SCN5A mutations to interpret clinically observed phenotypes and measured I_Na_ has been challenging [17,22], suggesting other modes of regulation.

The regulation of normal Nav1.5 expression is a complex event that involves multiple components of accessory and regulatory proteins, as well as the presence of other interacting ion channels [23] for trafficking to and stabilizing on the plasma membrane. Additionally, several endogenous microRNAs were discovered to post-transcriptionally regulate Nav1.5 expression [24]. These render the expression of Nav1.5 inferred by SCN5A transcript level using conventional reverse-transcription quantitative PCR (RT-qPCR) less suitable. For example, in haploinsufficient SCN5A^+/−^ mice, the mRNA level of SCN5A was similar to that in wild-type mice, yet the Nav1.5 level and the I_Na_ were both significantly reduced. In addition, conflicting results have been observed from qPCR, potentially due to different controls [25,26]. One solution to inconsistent results is to measure the absolute quantity as opposed to the relative quantity, in which a control is needed. Digital PCR (dPCR) has been used to measure the absolute amount of SCN5A mRNA [27]. Such results enabled cross comparison but were confined to transcript measurements. Given the importance of the Nav1.5 level to cardiac health, an accurate and direct measurement of the Nav1.5 protein quantity is critical for the diagnosis and treatment of relevant cardiac diseases.

However, there are no assays that can accurately measure Nav1.5 protein absolute expression. Electrophysiology can record the single channel and whole cell I_Na_ to estimate the number of functional sodium channels [28]. Nevertheless, it has been very difficult to carry out these measurements on human cardiomyocytes isolated from cardiac tissue due to ethical and technical challenges [29]. Immunoassays, such as Western blotting and fluorescence imaging, can provide relative quantities in animal and cell model studies [3,25,30], in which suitable controls are available. Nevertheless, a direct analysis of human tissue samples is still highly desired. Furthermore, relative quantities are incapable of comparing results in different patients across different labs due to varied controls. In addition, the relative quantitation of Nav1.5 prohibits its comparisons to other proteins, such as other ion channels and regulatory proteins that form complexes with Nav1.5 and regulate Nav1.5 trafficking. As a result, the lack of quantity in Nav1.5 expression has hindered a better understanding of Nav1.5 in diverse cardiac diseases.

To develop a quantitative assay that reports the copy number of Nav1.5 in both animal and cell models as well as in human tissues, here, we present a mass spectrometry (MS)-based targeted protein assay [31]. Targeted MS analysis can be achieved through selective reaction monitoring (SRM), multiple reaction monitoring (MRM), and parallel reaction monitoring (PRM) [32]. All these modes of MS analyses require a specific peptide as a surrogate to infer the target protein expression. Among the three, the PRM assay is the most suitable for initial screening of the surrogate peptide (s) and their associated transition (s) (precursor/product pairs) that can be implemented in SRM and MRM assays later. Here, we describe our development of a PRM assay for the absolute quantification of Nav1.5. We optimized the trypsin digestion, selected the surrogate reference peptides, screened suitable transitions, and characterized the lower limit of quantification (LLOQ). To obtain proof of principle, we applied our assay to study Chinese hamster ovary (CHO) cells transiently transfected with SCN5A.

## 2. Results

### 2.1. Trypsin Digestion

**Figure 2:**
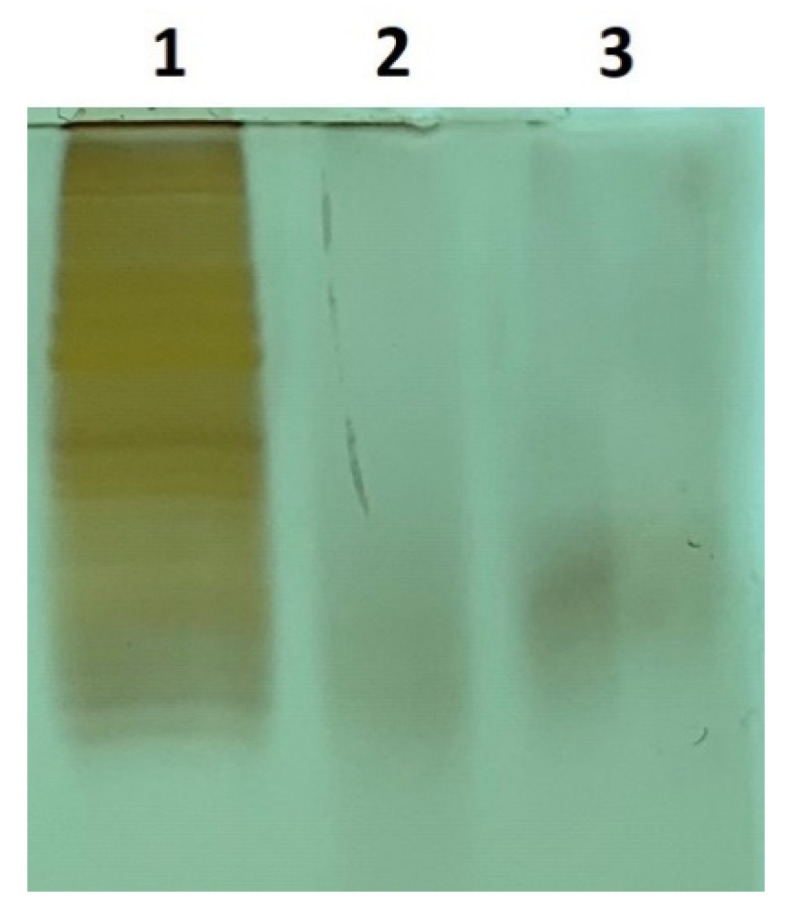
SDS-PAGE gel results of SCN5A transfected CHO cell lysates pre and post-trypsin digestion. Lane 1, pre-digestion; lane 2, 24-h post-digestion; lane 3, 48-h post-digestion.

We used a published trypsin digestion protocol [33,34] and monitored digestion efficiency overnight and at 24 and 48 h. The SDS-PAGE gel results of CHO cell lysate digestion at 24 and 48 h are shown in Figure 2. No obvious difference was observed among all the time points chosen. DDA-based proteomics analysis was carried out to evaluate the digestion efficiency. The mean miscleavages per peptide in the identified peptide groups and spectra mapped to peptides (PSMs) were 0.17 and 0.18, respectively, for the 24-h condition and 0.15 and 0.16 for the 48-h condition. Slight improvement was shown in the results of 48 over 24 h without statistical significance. Therefore, an incubation of 24 h was chosen as the condition for trypsin digestion.

### 2.2. LLOQ of Selected Peptides and Their Corresponding Transitions

Targeted MS quantification can achieve high specificity, accuracy, and multiplexity. Here, we used the PRM approach to examine four tryptic Nav1.5 peptides, as shown in Table 1, for their sensitivity to be used as surrogate targets to quantify the Nav1.5 protein in biological samples.

**Figure 3:**
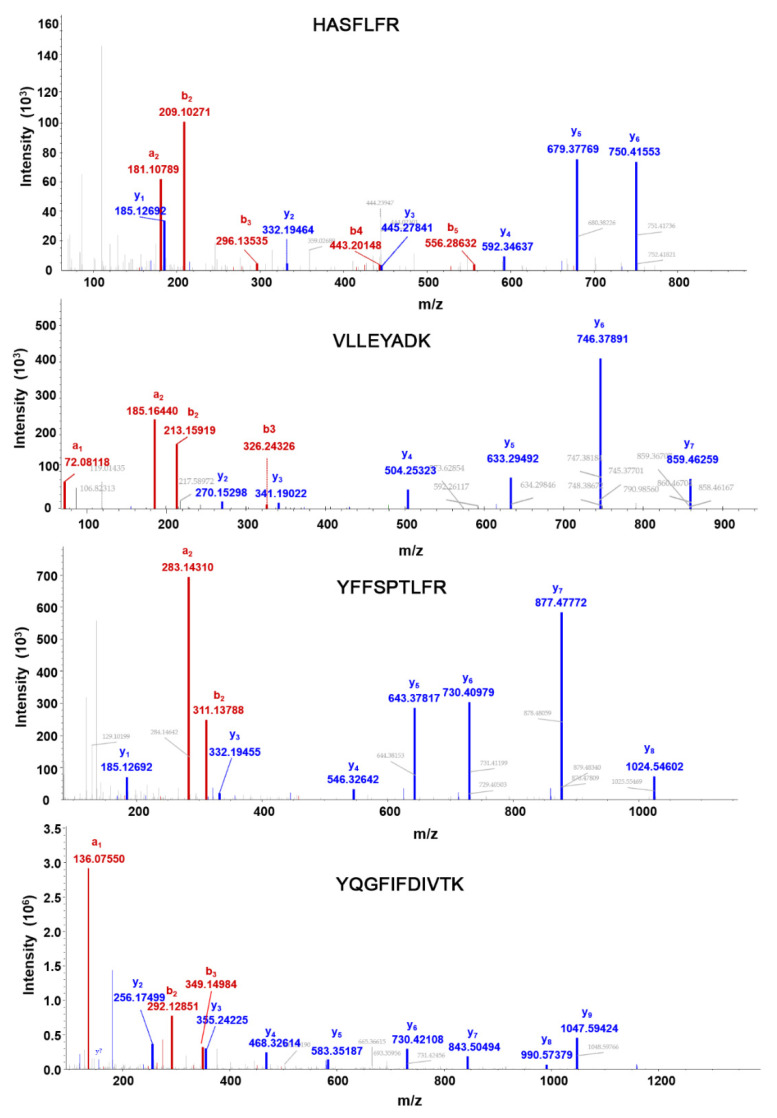
MS2 fragmentation patterns of Nav1.5 peptides (Heavy form). Red color denotes amino-terminal fragments, and blue color denotes carboxy-terminal fragments.

**Figure 4:**
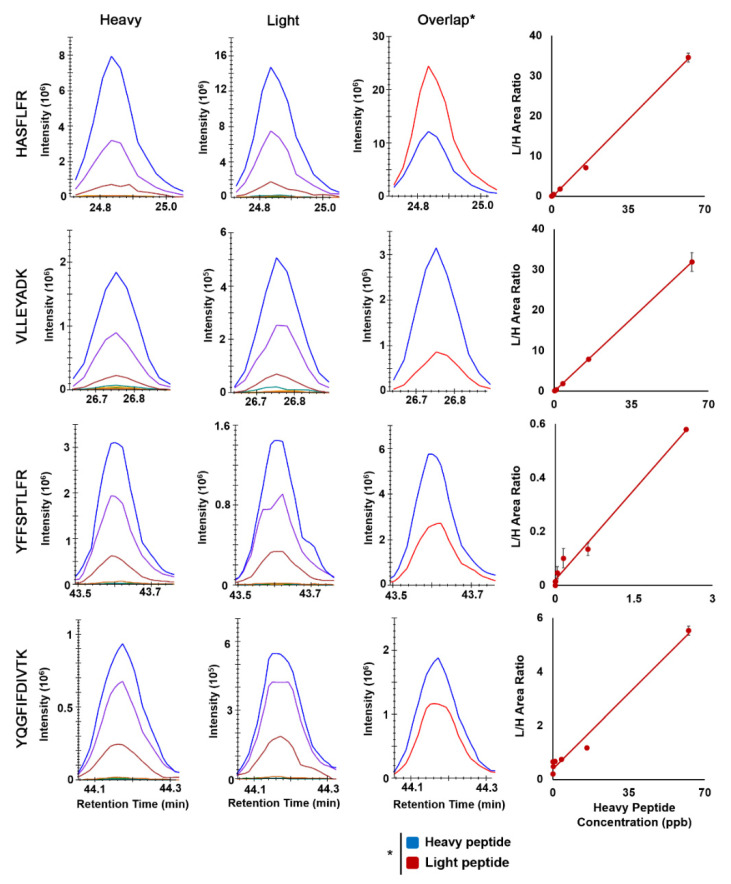
Skyline extracted chromatograms of heavy and light peptides of Nav1.5, the example overlap chromatograms of a particular transition, and their corresponding calibration curves. Linear least squares regression analysis was used to obtain the fitted calibration curve, and the dot is the mean and the error bar is the standard derivation of 3 replicates.

The MS2 fragmentation patterns of the selected peptides (heavy form) in the HCD (higher energy C-trap dissociation) cell of the Q-Exactive HF are shown in Figure 3. The top four abundant fragments derived from each peptide (light form) and their corresponding collision energies are listed in Table 1. To characterize the assay performance, we developed external calibration curves, in which light surrogate peptides with varied concentrations and heavy SIL peptides of a fixed concentration were spiked into control CHO cell samples that were not transfected with the SCN5A construct. The absence of Nav1.5 protein in the sample was confirmed by DDA MS analysis and the corresponding database search. For PRM analyses, extracted ion chromatograms of the light and heavy peptide transitions are shown in Figure 4.

To build the external calibration curve, seven different concentrations of light surrogate peptides were used plus the blank, in which only heavy SIL peptides with no light surrogate peptides were present in the control CHO cell samples. The calibration curve was developed for each transition, including both the parental precursors of M, M + 1, and M + 2 as well as the four daughter ions (fragments). We repeated such LC-MS/MS analysis three times to derive three external calibration curves for accessing the degree of variation, lower limit of quantitation (LLOQ) and assay robustness. First, we obtained the averaged calibration curves from three replicates, as shown in Figure 4. The standard deviations of the blank were used to compute the LLOQs of each transition, which are listed in Table 1.

### 2.3. Robustness of the Assay

**Figure 5:**
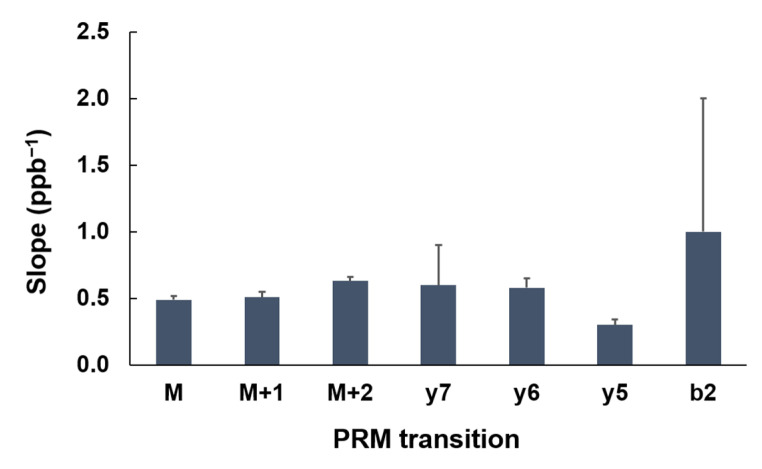
Slope variations among different transitions of VLLEYADK. The results are plotted as mean ± standard deviation, and the number of repeats is three. One-way ANOVA was performed for significant differences, and *p* value was greater than 0.05.

To evaluate the robustness of the assay. We repeated the calibration curve on three different dates. The slopes of the 3 replicates and the corresponding R^2^ values are listed in Table 2. The coefficient of variation (CV) of these slopes from each transition was used to assess the reproducibility and robustness of the assay. It is interesting to discover that the precursors in general produced lower CVs than those of the fragmented ions, as shown in Table 2. For example, for VLLEYADK, the CVs of the calibration-curve slopes of M, M + 1, and M + 2 were 0.06, 0.08, and 0.05, respectively. However, the CVs of the slopes of Y7, Y6, Y5, and B2 were 0.5, 0.1, 0.1, and 1, respectively. Nevertheless, the average slope of the three calibration curves among all transitions of the same peptide were similar to each other with no statistically significant difference (*p* > 0.05) based on one-way ANOVA, as shown in Figure 5.

### 2.4. Quantitation of SCN5A in Transiently Expressed CHO Cells

CHO cells transiently transfected with the SCN5A gene and its β1 subunit [35] were used to quantify endogenous Nav1.5 expression. To measure the Nav1.5 absolute quantity, we spiked heavy SIL peptides at suitable concentrations that were both below and above endogenous Nav1.5 peptides derived from SCN5A-transfected CHO cell lysates. In total, 1.5 million cells were used for analysis, of which five different spiking concentrations of heavy peptides were tested. With three repeated measurements of each condition, the obtained Nav1.5 expression was 3 ± 2 fmol on the column and 3 ± 2 × 10^4^ copies/cell in the sample.

## 3. Discussion

Sodium ion channels are critical in initiating cardiac action potentials, and their expression is finely controlled in healthy individuals [8,15]. Lethality along with various cardiac syndromes have been observed for patients with altered expression of the Nav1.5 protein, which is the channel-forming α subunit [5,6,8,9,15]. However, no assay was able to measure the absolute expression of Nav1.5 protein. The development of targeted MS assays on Nav1.5 poses unique technical challenges. The protein is ~220 KD and has four multipass transmembrane homologous domains (DI to DIV). Each of the four homologous domains has six transmembrane passes. Together, the protein harbors 24 transmembrane passes with relatively small extracellular but large intracellular regions. In addition, the protein carries many posttranslational modifications (PTMs), such as phosphorylation and glycosylation.

Only relative quantities of Nav1.5 have been reported in the literature using patch-clamp, Western blot, and immunofluorescence imaging [27,36,37]. Because relative quantitation requires a suitable control, these analyses were typically carried out in animal and cell models. From animal studies of SCN5A mutations, it has been clear that the expression level of mutants can affect the severity of phenotypes [10,38,39]. In humans, inherited SCN5A gene defects are often observed in aged populations, suggesting complex regulation. An accurate and absolute quantitation of Nav1.5 expression levels in human tissues will help provide valuable information that aids physiological and pathophysiological understanding of this crucial ion channel. An absolute quantity is usually more difficult to obtain than the relative quantity because the absolute quantity (copy numbers) often requires a calibration curve established with a known number of suitable references [40]. However, the added advantage of absolute quantitation is its broad availability for data comparisons and data integration among different studies.

Here, we developed an absolute quantification assay directly measuring protein expression with a set of synthetic stable heavy isotope-coded reference peptides of known concentrations. Using PRM mode, we screened four selected tryptic peptides and 28 transitions for their sensitivity and suitability as surrogate peptides for quantifying the expression of Nav1.5. Our results suggest that two peptides, i.e., VLLEYADK and HASFLFR are more sensitive than YFFSPTLFR and YQGFIFDIVTK in quantitation, as listed in Table 1.

When selecting all transitions above the corresponding LLOQs for the two most sensitive peptides, the obtained quantity of Nav1.5 in CHO cells was 3 ± 2 fmol on the column and 3 ± 2 × 10^4^ copies/cell. By patch-clamp techniques, the copy number of Nav1.5 per cell can be estimated by the total current recorded through whole-cell clamp and divided by the single-channel current. Using such estimation, we found that the copy numbers of Nav1.5 co-expressed with its β1 subunit in a reported overexpression system were in the range of 500–25,000 copies/cell [7,26,35,41,42,43,44]. Our results were at the upper level of this range, which may be because we measured the total expressed proteins including those in the secretory pathway or recycled from the plasma membrane. In addition, the transient expression of ion channels in vitro is prone to high expression [45]. Normally, only a small percentage of cells (~20%) have suitably low expression levels for whole-cell or single-channel patch clamping in transient expression systems [45]. Together, our results are congruent with the literature reports on the known expression level of Nav1.5.

To our knowledge, this is the first absolute quantitation of Nav1.5 in biological samples. We hope this assay can enable researchers to report copy numbers of this protein in their samples instead of measuring the increase or decrease in protein expression relative to the controls. Given the difficulty and scarcity of human tissue studies, the comparability of all obtained results enabled by absolute quantity will make the best use of these valuable results and avoid future unnecessary repeats. More importantly, such absolute quantitation by targeted MS is capable of the multiplexed analysis of hundreds of different proteins simultaneously and rapidly. The integration of the expression levels of different ion channels with other associated proteins permitted by such absolute quantitation will meet the demand of a data-driven bioinformatic and computer modeling era that will help to achieve predictive and preventative cardiac health and human health in general in the future.

## 4. Materials and Methods

### 4.1. Materials

Tris(2-carboxyethyl)phosphine (TCEP), dithiothreitol (DTT), iodoacetamide (IAA), and protease inhibitor cocktail were obtained from Sigma-Aldrich (St. Louis, MO, USA). Trypsin for cell culture was obtained from Corning (New York, NY, USA). Stable isotope-labeled (SIL) heavy tryptic peptides of Nav1.5 with ^15^N and ^13^C incorporated into either arginine or lysine were synthesized by JPT Peptide Technologies (Berlin, Germany), and the corresponding light-form surrogate peptides were synthesized by GenicBio Limited (Shanghai, China). The remaining chemicals without specification were obtained from Thermo Fisher Scientific, Waltham, MA, USA.

### 4.2. Cell Culture and Harvesting

Control Chinese hamster ovary (CHO) cells were maintained in sterile F12 (Ham’s) nutrient medium, pH 7.4 (Life Technologies, Thermo Fisher Scientific, Waltham, MA, USA), supplemented with 10% FBS (Life Technologies) and maintained in a humidified environment at 37 °C with 5% CO_2_ (Symphony 5.3A, VWR, Radnor, PA, USA) until 90% confluency. The CHO cells were transiently cotransfected with human cDNA encoding the Nav1.5 α-subunit, the β1-subunit, and eGFP following the PolyFect (Qiagen, Germantown, MD, USA) transfection protocol [33]. Transfected cells were grown in filtered sterile F12 (Ham’s) nutrient medium, pH 7.4 (Life Technologies, Thermo Fisher Scientific, Waltham, MA, USA), supplemented with 5% FBS and maintained in a humidified environment at 37 °C with 5% CO_2_ [46]. Confluent cell culture plates were rinsed 3 times with PBS and incubated with versene (0.5 mM EDTA, 1.1 mM d-glucose in PBS) at 37 °C for 10 min before being harvested by scraping. The cell pellet was formed by centrifuging the collected solution at 300–500 g and further rinsing with PBS.

### 4.3. Digestion

The cell pellets were dissolved and further digested following a previous protocol with slight modifications [33,34]. Typically, the cell pellet was first dissolved in denaturing solution (10 mM EDTA and 0.2% SDS in 40 mM Tris at pH 8.0) and boiled for 10 min. The protein amount was quantified by a BCA assay (Life Technologies, Thermo Fisher Scientific, Waltham, MA, USA). Then, TCEP was introduced at a final concentration of 10 mM, followed by adding powdered urea to the cooled sample to 8 M, and the solution was incubated at 37 °C for 30 min with end-to-end rotation. Disulfide bond formation was prevented by alkylating the cysteine residues with 15 mM iodoacetamide (IAA) in the dark at room temperature with an end-to-end rotation for 30 min. The reaction was halted with the addition of 20 mM DTT at room temperature for 15 min. The alkylated sample was diluted 10-fold with PBS prior to trypsin (Pierce, Cat. 90057, Thermo Fisher Scientific, Waltham, MA, USA) addition at a 1:20 enzyme-to-protein ratio. The digestion took place at 37 °C with end-to-end rotation overnight or for defined hours. The efficiency of digestion was evaluated by silver staining with 15% SDS-PAGE. The digested samples were further cleaned by an MCX column (Waters, MA, USA) and dried by a SpeedVac (Thermo Fisher Scientific, Waltham, MA, USA).

### 4.4. LC-MS/MS Analysis

Dried and digested control CHO samples were resuspended in MS loading buffer (1% acetonitrile and 0.1% formic acid (*v/v*) in HPLC water) and were spiked in both surrogate light and SIL heavy peptides at various concentrations for external calibration. SCN5A-transfected CHO samples were spiked in SIL heavy peptides alone at various concentrations for internal calibration. An EASY-nLC 1000 system was coupled to Q-Exactive HF through the nano-EASY spray source (all from Thermo Scientific, Mississauga, ON, Canada) for the subsequent nanoLC-MS/MS analyses. Peptides (0.5 µg) were loaded first onto a Pepmap 100 trapping column (Thermo Scientific, 20 mm × 75 μm ID, 3 μm C18 resin with 100 Å pore size) before they were eluted on a Pepmap EASY-spray analytical column (Thermo Scientific, 150 mm × 75 μm ID, 3 μm C18 resin with 100 Å pore size) with an integrated spray tip. A 60 min gradient of 2–35% acetonitrile in 0.1% formic acid (*v/v*) at a constant flow rate of 300 nL/min was used to elute peptides off the analytical column.

For data-dependent acquisition (DDA), Q-Exactive HF MS2 scan parameters were set to select the top 10 most abundant precursors in MS1 scans for higher energy collisional dissociation (HCD) with a dynamic exclusion of 10 s. MS1 scans were acquired at the m/z range of 400–2000, mass resolution of 120,000, automatic gain control (AGC) target of 3 × 10^6^, and maximum ion injection time of 100 ms. The threshold to trigger MS2 scans was 1.0 × 10^3^, the normalized HCD energy was 22%, and the resolved fragments were scanned at a mass resolution of 30,000 and an AGC target value of 5 × 10^5^. Precursors with changes of 1, 5–7, >8, and unassigned were excluded from MS2 scans. The max ion injection time for MS2 scans was 100 ms, and the isolation window was 2.0 Th.

For parallel analysis monitoring (PRM), Q-Exactive HF MS2 scan parameters were set to select the m/z ratio of endogenous and the corresponding SIL peptides of Nav1.5 from the inclusion list. The MS1 scans were acquired in the m/z range of 400–2000 with a mass resolution of 120,000, automatic gain control (AGC) target of 3 × 10^6^, and maximum ion injection time of 200 ms. The PRM scans were acquired at a resolution of 30,000, AGC target value of 2 × 10^5^, maximum ion injection time of 150 ms, isolation window of 2.0 Th with 0.5 Th offset, and the normalized HCD energy between 22% and 27%.

### 4.5. Data Processing

For DDA, the resolved raw files from Q-Exactive HF were processed by Proteome Discoverer 2.1 (Thermo Scientific, Mississauga, ON, Canada) and searched using Sequest HT. A default signal-to-noise threshold of 1.5 was applied to the MS2 spectra. The search was against the mouse UniProt database appended with common contaminating sequences (trypsin and keratin) [47]. The parameters of the search were 10 ppm precursor mass tolerance (monoisotopic mass), full tryptic ends with 2 missed cleavages, minimum 6 amino acids, and 0.05 Da fragment mass tolerance (monoisotopic mass). Cysteine carbamidomethylation was treated as a fixed modification, and methionine oxidation, deamidation of asparagine, and N-acetylation of protein were treated as dynamic modifications. The Decoy database was used for false discovery rate (FDR) estimation (strict FDR cutoff: 0.01), and Percolator was used to filter false positives. The final result was filtered against a peptide confidence level of 0.01 FDR (high), and protein groups were considered as the protein identification in which congeneric proteins identified by the same peptides were grouped into one.

For PRM, Skyline was used for processing the data [48]. The spectral library of the surrogate peptides was built in Skyline based on DDA MS analysis of the synthetic peptide mixture of both heavy and light peptides. Peak picking and peak boundaries were carried out by Skyline and manually adjusted based on the overlapping precursor and product peaks. The peak area for each transition was exported to Microsoft Excel. To build the calibration curve, the ratios between light and heavy peptides were plotted against the concentrations of SIL heavy peptides.

For data analysis, linear least squares regression analysis was applied using Microsoft Excel to obtain the fitted calibration curve. The goodness of fitting was evaluated by the correlation coefficient (R), expressed as the coefficient of determination (R^2^). The lower limit of quantitation (LLOQ) is estimated based on 10 * σ/m, in which σ is the standard deviation at the minimum concentration in the calibration curve and m is the slope of the curve. Each calibration curve was repeated three times. The coefficient of variation (CV) was computed for the obtained slopes and R^2^. One-way ANOVA in Microsoft Excel was also applied to analyze the significant changes among slopes of MS1 precursors and MS1/MS2 transitions in the same peptide, and a *p* value < 0.05 was considered significant.

## Figures and Tables

**Table 1:** PRM peptides of Nav1.5.

Peptide Sequence	MS1 Light M.W. (g/mol)	MS2 Type	MS2 Light M.W. (g/mol)	Neutralized CE * (%)	LLOQ ** Light (fmol)
HASFLFR	878.4752	precursor	878.4752		4
		precursor [M + 1]	879.4780		5
		precursor [M + 2]	880.4807		7
		y6	740.4090	27	2
		y5	669.3719		1
		a1	110.0713		2
		b2	209.1033		3
VLLEYADK	951.5266	precursor	951.5266		0.4
		precursor [M + 1]	952.5296		0.08
		precursor [M + 2]	953.5323		2
		y7	851.4509	22	0.2
		y6	738.3668		0.2
		a2	625.2828		0.3
		b2	213.1598		0.4
YFFSPTLFR	1178.6113	precursor	1178.6113		8
		precursor [M + 1]	1179.6144		0.7
		precursor [M + 2]	1180.6172		0.3
		y7	867.4723	27	2
		y6	720.4039		0.3
		y5	633.3719		0.3
		b2	311.1390		0.8
YQGFIFDIVTK	1331.7114	precursor	1331.7114		5
		precursor [M + 1]	1332.7145		2 × 10
		precursor [M + 2]	1333.7173		4
		y9	1039.5823	27	1
		y6	722.4083		4
		a1	136.0757		6
		b2	292.1292		0.1

* collision energy, ** LLOQ stands for lower limit of quantitation.

**Table 2:** Calibration curve properties of all selected peptides.

		HASFLFR		VLLEYADK		YFFSPTLFR		YQGFIFDIVTK	
Transition		Slope	R^2^ ****	Slope	R^2^	Slope	R^2^	Slope	R^2^
M	avg. *	0.55	0.998	0.49	0.998	0.2	0.981	0.076	0.958
	stdev. **	0.02	0.0005	0.03	0.001	0.01	0.008	0.005	0.01
	CV ***	0.036	0.0005	0.061	0.001	0.05	0.0082	0.066	0.01
M + 1	avg.	0.66	0.997	0.51	0.9995	0.211	0.989	0.088	0.964
	stdev.	0.08	0.002	0.04	0.0005	0.009	0.008	0.003	0.002
	CV	0.12	0.002	0.078	0.0005	0.043	0.0081	0.034	0.0021
M + 2	avg.	0.64	0.998	0.63	0.999	0.233	0.981	0.096	0.966
	stdev.	0.07	0.0025	0.03	0.0004	0.004	0.016	0.001	0.008
	CV	0.11	0.0025	0.048	0.0004	0.017	0.016	0.01	0.0083
y9	avg.							0.092	0.918
	stdev.	N/A		N/A		N/A		0.05	0.1
	CV							0.54	0.11
y7	avg.			0.6	0.989	0.12	0.963	0.09	0.972
	stdev.	N/A		0.3	0.01	0.04	0.04	0.04	0.027
	CV			0.5	0.01	0.33	0.042	0.44	0.028
y6	avg.	0.6	0.989	0.58	0.996	0.20	0.957	0.2	0.8695
	stdev.	0.3	0.01	0.07	0.004	0.04	0.024	0.3	0.2
	CV	0.5	0.01	0.12	0.004	0.2	0.025	1.5	0.23
y5	avg.	1.4	0.991	0.3	0.988	0.14	0.95		
	stdev.	0.7	0.004	0.04	0.01	0.04	0.036	N/A	
	CV	0.5	0.004	0.13	0.01	0.29	0.038		
y1	avg.	0.8	0.993						
	stdev.	0.4	0.005	N/A		N/A		N/A	
	CV	0.5	0.005						
b2	avg.	1	0.97	1	0.981	0.14	0.976	0.1	0.976
	stdev.	1	0.015	1	0.014	0.05	0.034	0.1	0.0044
	CV	1	0.016	1	0.014	0.38	0.035	1	0.0045

* avg. stands for arithmetic mean, ** stdev. stands for standard deviation, *** CV stands for coefficient of variation, **** R^2^ stands for coefficient of determination.

## Data Availability

Not applicable.
